# Serum C-reactive Protein Levels about Insulin Resistance and Beta Cell Function in Iranian Women with Subclinical Hypothyroidism

**DOI:** 10.30699/ijp.2024.2015823.3213

**Published:** 2024-07-24

**Authors:** Amirali Ayatollahi, Zohre Moosavi, Hossein Ayatollahi

**Affiliations:** 1 Cancer Molecular Pathology Research Center, Mashhad University of Medical Sciences, Mashhad, Iran; 2 Endocrine and Metabolism Research Center, Mashhad University of Medical Sciences, Mashhad, Iran

**Keywords:** Beta cell function, C-reactive protein, Insulin resistance, Subclinical hypothyroidism

## Abstract

**Background & Objective:**

Subclinical hypothyroidism (SCH), is defined as an asymptomatic state characterized by a normal serum concentration of free thyroxine and elevated serum concentration of TSH. This study aims to investigate the complex interplay between hyperinsulinism, insulin resistance, beta cell function, and low-grade chronic inflammation in Iranian women with SCH.

**Methods:**

Eighty women with SCH and 80 healthy women as controls matched to the patient group for sex, age, and body mass index (BMI), were enrolled in this prospective cross-sectional study. TSH, free T3, free T4, highly sensitive C-reactive protein (hs-CRP), fasting insulin, fasting glucose, total cholesterol, HDL-cholesterol, LDL-cholesterol, Triglyceride, HOMA-IR index, Beta cell function (HOMA-B index) and insulin sensitivity were determined.

**Results:**

Hs-CRP was not statistically different between the SCH patients and the control group (3.7±3.2 Vs 3.6±4.0, *P*>0.05). Total cholesterol and LDL-cholesterol were significantly higher; however, triglyceride and HDL-cholesterol were not statistically different in patients with SCH as compared with the control group. Fasting insulin levels and HOMA-IR and Beta cell function (HOMA-B) were significantly higher in SCH women compared to the control group. A positive correlation between HOMA-IR and HOMA-B with TSH levels was found (r=0.324, r=0.191, *P*<0.05 respectively). A positive correlation between insulin levels and hs-CRP (r=0.22, *P*<0.05), also between insulin levels and TSH (r=0.312, *P*<0.05) and LDL- LDL-cholesterol was obtained (r=0.27, *P*<0.05).

**Conclusion:**

Iranian women with SCH may exhibit elevated atherogenic parameters (hyperinsulinemia, LDL-cholesterol, and total cholesterol), HOMA-IR, and HOMA-B.

## Introduction

Subclinical hypothyroidism (SCH) refers to a state in which the serum thyrotropin level is higher than the upper limit of the reference interval, while the serum free thyroxine (FT4) is normal (1-3) may be caused by exogenous (L-thyroxin under- replacement in hypothyroid patients, medications with lithium, cytokines, iodine, or antithyroid drugs, and iodine 131 therapy or thyroidectomy) or endogenous (Hashimoto thyroiditis and previous subacute or silent thyroiditis) factors (4). In two population-based studies, the prevalence of subclinical hypothyroidism ranged between 7.5–8.5% in women and 4.4% in men (5, 6). Subclinical hypothyroidism prevalence increases in women with increasing age and is more common in elderly females (7–18%) than males (2–15%) (7, 8). Epidemiologic studies have shown that, in elderly persons, subclinical hypothyroidism is associated with an increased risk for atherosclerosis and coronary artery disease (9). In addition, few studies have investigated the relationship between coronary heart disease and SCH. Knowing this association can be valuable in predicting the development of coronary heart disease in SCH patients. Necropsy studies confirmed this association for subclinical hypothyroidism (10). Furthermore, since atherosclerosis is presumed to be an inflammatory disease, C-reactive protein (CRP), as determined by a highly sensitive immunoassay, has been suggested as a strong predictor of cardiovascular risk (11, 12). Some studies report that moderate elevation of CRP (below those of most routine CRP assay) correlates with future cardiovascular events validating the use of this test to assess cardiovascular risk (13). To identify these moderate elevations, the performance of high-sensitivity assays for CRP (hs-CRP) is recommended to better identify CRP variations. Thyroid hormones influence glucose homeostasis. The insulin resistance syndrome has characteristics of endocrine and metabolic disturbance along with inflammatory and prothrombotic states (14). Some reports suggested a positive association between components of insulin [resistance syndrome and CRP (15). The Insulin Resistance Atherosclerosis Study (IRAS) showed that insulin resistance was significantly related to higher CRP levels (16). The aim of this study is to investigate the complex interplay between insulin resistance, Beta-cell function, and low-grade chronic inflammation such as hs-CRP with subclinical hypothyroidism in Iranian women.

## Material and Methods

**A-Subjects**

Eighty women with SCH (mean age 33.2±5.0 years) were included in the study. Eighty age and BMI-matched euthyroid healthy hospital staff were selected as the control group (mean age 35.1±4.2). Histories, physical examination, chest radiography, ECG, and routine chemical analysis showed that the control subjects had no evidence of any disease. Diabetic subjects, alcohol users, smokers, those with pituitary/hypothalamic disorders, those taking thyroid hormone medication up to 3 months before enrollment, and drugs known to affect TSH, CRP, and lipid levels were excluded from the study. All patients were recruited from the endocrine and metabolism research center at Mashhad University of Medical Sciences, Mashhad, Iran. Some of the patients were referred to this center for obesity workup, malaise, stress disorders, and other nonspecific symptoms. All patients gave informed voluntary consent to participate in the study according to the protocol approved by the local ethics committee of MUMS and in accordance with the ethical standards of the Helsinki Declaration Conditions that could be associated with an acute-phase reaction or patients with a history of acute myocardial infarction or angioplasty within the preceding 6 months and patients diagnosed with the concomitant disease (arthritis) were also excluded. Patients with a history of smoking and past thyroid disorders or any therapy were also excluded. The inclusion criteria for SCH patients were as follows: 20-40 years old, premenopausal stage, TSH level more than 5mIU/L on two consecutive blood tests, freeT4 and freeT3 concentrations within the normal range and good general health as assessed by a full medical and endocrine work up.

**B-Methods:**

**Anthropometric Measurement**

Body weights (Kg) with light clothing heights (cm) were measured without shoes and/or caps. Height was recorded to the nearest centimeters and weight to the nearest 0.1 kg Body mass index (BMI) was expressed as weight per height square (kg/m2)

**Biochemical Evaluation**

All blood samples were taken between 08.00-09.00 AM after 12 hours of fasting period. After collection and immediately centrifugation in 1500g for 15 min serum samples were stored at -20°C until assayed. Total cholesterol, HDL-cholesterol, LDL-cholesterol, triglyceride, and glucose levels were determined with a biochemistry autoanalyzer. (Liasys, Rome, Italy). Serum fasting insulin, Serum TSH levels, fT3, and fT4 levels were measured by electrochemiluminescence method with Cobas e 411analyzer (Roche Company-Germany). Serum CRP levels were measured with immunoturbidimetric assay by high sensitivity CRP kit (Diagnostica, Germany). The intra-assay CV and inter-assay CV for hs-CRP were found 5.2% and 3.3%

**Assessment of Insulin Resistance and Beta-cell Function**

Insulin resistance and Beta-cell function were also calculated using homeostatic model assessment (HOMA) (17). HOMA uses a structural model of glucose-insulin interactions to describe the responses of major organs (17) and allows evaluation of the specific combination of deficient beta-cell function and impaired insulin sensitivity provided by the basal glucose and insulin concentrations in a patient (17) HOMA-derived insulin resistance (HOMA-IR) was calculated as follows: fasting insulin (µu/mL) × fasting plasma glucose (mmol/ml)/22.5 HOMA-derived beta-cell function (HOMA-B) was also calculated using the. following formulae: 20×fasting insulin (µu/ml)/fasting glucose (mmol/ml)-3.5 (17)

**4: Statistical analysis**

All values were expressed as mean±SD (standard deviation) SPSS 19 (SPSS-Inc Chicago, IL, USA) for Windows 10 used for Statistical analysis. An Independent t-test was used to compare patients with SCH and the control group.

Pearson correlation test was used to define the correlation among hs-CRP, TSH, fasting insulin, HOMA-IR, and HOMA-B values. P-values <0.05 were accepted as statistically significant.

## Results

**1: Baseline Characteristic**

When the clinical characteristics of 80 Iranian women of SCH and 80 control persons were compared, no significant differences were observed according to age, BMI, systolic and diastolic blood pressure (Table 1)

**2: Biochemical Characteristic**

Fasting plasma glucose, triglycerides, fT3, fT4, and HDL-cholesterol concentrations in SCH and the control group did not differ (Table 2). Total and LDL-cholesterol levels of subjects with SCH were higher than the control group (Table 2). Mean TSH levels of subjects with SCH were significantly higher than control subjects (9.3± 3.2µIu/ml and 1.6±0.8µIu/mL, respectively). Patients with SCH were not different from the control group in terms of fT3 and fT4 levels (Table 2). Mean insulin, HOMA-IR and HOMA-B values of subjects with SCH were also significantly higher concerning control subjects (Table 2). Patients with SCH were not different from the control group in terms of hs-CRP (3.7±3.2 vs. 3.6±4.0, P>0.05). (Table 2). We performed a Pearson correlation test among hs-CRP, TSH, total cholesterol, and LDL-cholesterol with insulin levels, HOMA-IR, and HOMA-B values. We found a positive correlation between insulin levels and hs-CRP (r=0.22, *P*<0.05), TSH levels (r=0.312, *P*<0.05), LDL-cholesterol (r=0.27, *P*<0.05) HOMA-IR values were not correlated with hs-CRP, total cholesterol and LDL-cholesterol (Table 3). HOMA-B and HOMA-IR were correlated with TSH in Iranian women with SCH (Table 3).

**Table 1:** Clinical characteristics of the patients with subclinical hypothyroidism and controls

	Subclinical Hypothyroidism(n=80)	Control (n=80)	P-value
Age (yr)	33.2±5	35.1±4.2	*P*>0.05 NS
BMI (kg/m2)	26.3±5.2	25.2±6.1	*P*>0.05 NS
Systolic blood	130.1±6.4	128.3±4.8	*P*>0.05
Pressure (mmHg)			NS
Diastolic blood	69.9±5.4	71.2±6.1	*P*>0.05
Pressure (mmHg)			NS

NS: Not significant

**Table 2:** Comparison of biochemical and hormonal parameters of the Iranian women with SCH and control women group.

	Subclinical Hypothyroidism(n=80)	Control (n=80)
TSH(µIu/ml)	9.3± 3.2	1.6±0.8 *
fT4(Pmol/L)	12.5± 6.5	14.0±3.5
fT3(Pmol/L)	3.9±3.6	3.6 ±1.5
Fasting glucose(mmol/L)	5.8±1.3	5.7±1.8
Fasting insulin(µu/mL)	18.8 ±11.2	12.6± 15.2 *
Total cholesterol(mmol/L)	6.47±1.2	5.8 ±1.0 *
LDL cholesterol(mmol/L)	4.2 ±1.4	3.5 ±1.0 *
HDL cholesterol(mmol/L)	1.5 ±0.2	1.4 ± 0.2 *
Triglyceride(mmol/L)	2.0±0.8	1.8±0.7
hsCRP(mg/L)	3.7±3.2	3.6± 4
HOMA-IR	5.0±1.7	3.5 ±1.5 *
HOMA-B	206.2±171.5	168.5± 76.98*

Significant P< 0.05 *

**Table 3:** Pearson correlation between insulin, HOMA-IR, HOMA-B%, and insulin sensitivity with TSH level, hs CRP, and LDL in the Iranian women with SCH.

Correlation	Insulin	HOMA-IR	HOMA-B
TSH	r = 0.312	r=0.324	r = 0.191
	*P*<0.05	*P*<0.05	*P*<0.05
hs-CRP	r = 0.22	NS	NS
	*P*<0.05	*P*>0.05	*P*>0.05
LDL	r = +0.27	NS	NS
	*P*<0.05	*P*>0.05	*P*>0.05

NS: Not significant

## Discussion

Whether subclinical hypothyroidism (SCH) is considered a risk factor for cardiovascular disease is controversial (18-21), although it has been concluded that mild thyroid failure is associated with an increased risk for development of atherosclerosis (22) and that SCH is a strong indicator for risk of atherosclerosis and myocardial infarction in elderly women (21). The Wickham survey found no relationship between initial TSH levels and the subsequent development of ischemic heart disease over 20 years of follow-up (23) but in the Rotterdam study, a serum TSH level greater than 4.0 µIu/mL was found to be associated with a history of myocardial infarction and atherosclerosis of the abdominal aorta (22). Thyroid hormones affect the synthesis, conversion, and removal of lipids (24) and increase the sensitivity of lipolytic hormones such as catecholamine for adipose tissue, in addition, thyroid hormones affect protein synthesis by controlling the expression of LDL-cholesterol receptor genes on cell surface and thus contribute to lipoprotein metabolism (25). Kutty* et al.* (26) examined the relationship between thyroid diseases and lipid metabolism by dividing thyroid diseases according to their degree into six groups based on TSH level and clinical symptoms; they found a correlation between thyroid function and cholesterol levels. However, they reported that serum cholesterol level is not a sensitive marker of metabolic change induced by thyroid hormones. Lee* et al.* found that the levels of serum total cholesterol and LDL-cholesterol were not significantly different between subclinical hypothyroidism and the control group (27). We did, however, find significant differences in total cholesterol and LDL-cholesterol between subclinical hypothyroidism and control groups. However, triglyceride and HDL-cholesterol were not statistically different. The same results were obtained and Al Sayed *et al.* (14). Researchers have focused on the role of inflammatory reactions as a pathogenic mechanism for atherosclerosis. Some studies have reported that CRP is increased in patients with atherosclerosis and that this is related to prognosis (25).

Studies on the role of CRP in the relationship between subclinical hypothyroidism and atherosclerosis have not been conducted. Cherist-Crain* et al.* found that serum CRP levels were significantly elevated in subclinical hypothyroidism versus normal controls However they found no significant correlation between CRP and TSH directly (28). Thus, they concluded that the association between thyroid status and CRP is, if present at all, weak (28). Lee* et al.* (27) and Al Sayed* et al.* (14) showed that hs-CRP was not significantly different between subclinical hypothyroidism and control groups. However, we found that there was no significant difference in hs-CRP between subclinical hypothyroidism and the control group. We also found no significant correlation between CRP and TSH in Iranian women with SCH. The same result was reported by Al Sayed (14) and Luboshitzky and Herer (29). This supports that CRP does not appear to contribute to the increase in ischemic heart disease in women with SCH. In our study, we found significantly higher insulin levels, HOMA-IR, and HOMA-B in the SCH group comparable to the control group. Dssein* et al.* (30) also found insulin resistance in patients with SCH, but Al Sayed did not report a significant difference in HOMA-IR between SCH and control groups. These reports could indicate ethnic differences in insulin resistance in SCH patients and probably Iranian women with SCH have a higher risk for diabetes. It is the first report of HOMA-B in SCH women in our literature Review. We also found a positive correlation between hs-CRP and insulin levels in female patients with SCH. This correlation showed that hs-CRP might induce an interaction with insulin in these patients. The Insulin Resistance Atherosclerosis Study (IRAS) (16) and Wu* et al.* in school children in Taiwan showed that insulin resistance was significantly related to higher CRP levels (31) but in our study, we did not find this correlation in the Iranian women with SCH. 

## Conclusion

In this study, we found that there was a significant positive correlation between HOMA-IR and HOMA-B with TSH in Iranian women with SCH. We showed there is insulin resistance in women with SCH and hyperinsulinemia and increased beta cell function in these patients may be due to insulin resistance. However, despite of significant difference in HOMA-IR and HOMA-B values in the Iranian women with SCH, there is no correlation of HOMA-IR with atherosclerosis risk factors such as hs-CRP, total, and LDL-cholesterol in these patients. In patients with SCH, there is a significant positive correlation between hyperinsulinism and atherosclerosis risk factors. We conclude that patients with SCH may exhibit elevated atherogenic parameters (hyperinsulinemia, LDL-cholesterol, and total cholesterol), HOMA-IR, and HOMA-B. 
